# Thyroid Dysfunction and Renal Function: A Crucial Relationship to Recognize

**DOI:** 10.7759/cureus.35242

**Published:** 2023-02-20

**Authors:** Rania Naguib, Eman Elkemary

**Affiliations:** 1 Internal Medicine Department, Endocrinology Unit, Faculty of Medicine, Alexandria University, Alexandria, EGY; 2 Clinical Pathology Department, Faculty of Medicine, Alexandria University, Alexandria, EGY

**Keywords:** kidney function, renal function, thyroid dysfunction, hyperthyroidism, hypothyroidism

## Abstract

Background and Aim: Renal function is noticeably altered in both hypothyroidism and hyperthyroidism. However, clinical studies on thyroid dysfunction and its association with renal function are scarce. The purpose of this study was to evaluate changes in biochemical parameters of renal function in subjects with thyroid dysfunction and to correlate these values with the patient's thyroid profile. The effect of changes in thyroid function during therapy on renal function was also investigated.

Methods: A prospective cohort study included 41 patients with untreated primary hypothyroidism and 16 patients with untreated hyperthyroidism. Thyroid-stimulating hormone (TSH), free thyroxine, and free triiodothyronine were assessed using immunoassay. The estimated glomerular filtration rate was calculated by the Modification of Diet in Renal Disease formula. Renal function tests were assessed in all patients at each of the two-time points: during thyroid dysfunction (hypo- or hyperthyroidism) and after attaining euthyroidism.

Results: Our study demonstrated a statistically significant reduction in the average serum creatinine level in the hypothyroid patients after treatment compared to before treatment whereas the mean estimated glomerular filtration rate (eGFR) significantly improved after treatment compared to before treatment. Moreover, the average serum creatinine level in the hyperthyroid patients was significantly lower before treatment compared to after treatment, whereas the mean eGFR significantly dropped after treatment. TSH had a significant positive correlation with serum creatinine and a significant negative correlation with eGFR in all patients with thyroid dysfunction.

Conclusions: Thyroid dysfunction is associated with deranged kidney function. It is crucial for the clinician to be aware of the link between thyroid disorders and aberrant renal function in order to consider a thyroid function test when treating a patient whose biochemical markers of renal function are only mildly elevated. There is a need for monitoring creatinine in patients with thyroid dysfunction.

## Introduction

Thyroid dysfunction has been proven to alter the function of all organ systems in the body such as the heart, muscles, and brain. Thyroid status has an impact on renal function as well. The thyroid hormone affects the kidney through systemic or local hemodynamic changes as well as a direct effect on its function [1]. Changes in renal blood flow, glomerular filtration rate (GFR), tubular secretory and absorptive capacity, electrolyte pumps, and kidney structure are all effects of thyroid dysfunction. Renal function is noticeably altered in both hypothyroidism and hyperthyroidism [2]. The association of various types of glomerulopathies with thyroid hyper- and hypofunction has been reported [3]. However, clinical studies on thyroid dysfunction and its association with renal function are scarce, and little is known about how thyroid dysfunction affects renal function in humans. Most studies on the effects of thyroid hormone on the kidney have been conducted in rats [2]. Furthermore, the renal effects of thyroid hormones in humans can be subtle, escaping clinical attention because changes in measured renal function parameters are frequently within the normal range.

The purpose of this study was to evaluate changes in biochemical parameters of renal function in subjects with thyroid dysfunction and to correlate these parameters with the patient's thyroid profile. The effect of changes in thyroid function during therapy on renal function was also investigated.

## Materials and methods

A prospective cohort study included 41 patients (33 females and eight males) with untreated primary hypothyroidism and 16 patients (14 females and two males) with untreated hyperthyroidism (In seven patients, it was caused by Graves' disease and in nine by toxic adenoma). Patients were recruited from the Internal Medicine Department, Alexandria Faculty of Medicine in the period between June and July 2022. The study was approved by the ethics committee at the Alexandria University Faculty of Medicine (Approval number: 00012098, Federalwide Assurance (FWA) number: 00018699). All participants provided written informed consent.

Full history and clinical examination were conducted to rule out any renal problems or other inflammatory conditions that might have affected the study's data. Patients who were concurrently taking medications that might impair renal function were excluded. Thyrotoxic patients received antithyroid medication or radioiodine to treat hyperthyroidism until their euthyroidism was restored. Thyroid hormone replacement therapy with levothyroxine was used to treat hypothyroid people. The patients were examined at the time of diagnosis and two to three months after attaining the euthyroid status, defined as free thyroxine level (FT4) or thyroid stimulating hormone (TSH) being within the reference range for individuals with hyperthyroidism or hypothyroidism, respectively.

Routine laboratory investigations

 TSH, FT4, and free triiodothyronine (FT3) were measured using COBAS E411 automated immunoassay analyzer (Roche Diagnostics, Germany). Renal function tests; creatinine, blood urea nitrogen (BUN), and albumin were measured using COBAS C311 automated chemistry analyzer (Roche Diagnostics, Germany) [4]. The estimated GFR (eGFR) was calculated by the Modification of Diet in Renal Disease (MDRD) formula. The MDRD equation has several limitations, one of which is that it is less accurate at levels greater than 60 ml/min per 1.73 m^2^. As a result, individuals with mild renal insufficiency may be misdiagnosed and misclassified as having chronic kidney disease (CKD) [5]. Renal function tests were assessed in all patients at each of the two time points: during thyroid dysfunction (hypo- or hyperthyroidism) and after attaining euthyroidism.

Statistical analysis

Statistical analysis was carried out using IBM SPSS Statistics for Windows, Version 20.0 (Released 2011; IBM Corp., Armonk, New York, United States). Data were expressed as means ± SD. The paired Student t-test was used to analyze values within the two groups before and after treatment. The Spearman test was utilized to calculate correlations. A p-value of < 0.05 was deemed significant.

## Results

In our study population, the majority of the patients were women (33 hypothyroid and 14 hyperthyroid women). The mean age for the hypothyroid patients was 38± 4 years while in hyperthyroid patients, the mean age was 33±5 years. The mean TSH level in hypothyroid patients (n = 41) was 11.1±3.7 mIU/L (reference 0.34-5.6 mIU/L) at diagnosis and decreased to 4.3 ±1.2 mIU/L when patients were treated with levothyroxine. There was a statistically significant reduction in the average serum creatinine level in the hypothyroid patients after treatment compared to before treatment (0.97 ± 0.32 versus 2.3 ±0.7 mg/dl; p-value P = 0.011) (reference 0.74-1.35 mg/dL for adult men and 0.59-1.04 mg/dL for adult women ), whereas the mean e-GFR significantly improved after treatment (79 ± 13 ml/min) compared to before treatment (67 ±11 ml/min) (normal range > 60 ml/ min) (p-value < 0·01) (Table 1).

**Table 1:** Comparison of thyroid and renal function parameters among hypothyroid patients before and after treatment of thyroid dysfunction TSH: thyroid-stimulating hormone; FT: free thyroxine; eGFR: glomerular filtration rate; MDRD: Modification of Diet in Renal Disease

	Before treatment	After treatment	p-value
TSH (mIU/L)	11.1±3.7	4.3 ±1.2	P < 0·001*
FT3 (pg/mL)	1.9 ± 0.5	3.1 ± 0.8	P < 0·01*
FT4 (pmol/L)	6 ±4.3	22 ±7	P = 0·003*
Creatinine (mg/dl)	2.3 ±0.7	0.97 ± 0.32	P = 0.011*
eGFR (MDRD)	67 ±11	79 ± 13	P < 0·01*

The mean FT4 level in hyperthyroid patients (n = 16) was 54 ± 11 pmol/l at diagnosis (reference 12- 30 pmol/L) and dropped to 21 ±8 pmol/l when patients were treated with antithyroid drugs or radioiodine. The average serum creatinine level in the hyperthyroid patients was significantly lower before treatment compared to after treatment (0.3 ±0.1 versus 1.1 ± 0.2 mg/dl; p-value < 0·03), whereas the mean eGFR significantly dropped after treatment (101 ± 11 ml/min) compared to (127 ±37 ml/min) before treatment (p-value < 0·01) (Table 2).

**Table 2:** Comparison of thyroid and renal function among hyperthyroid patients before and after treatment of thyroid dysfunction TSH: thyroid-stimulating hormone; FT: free thyroxine; eGFR: glomerular filtration rate; MDRD: Modification of Diet in Renal Disease

	Before treatment	After treatment	p-value
TSH (mIU/L)	0.1± 0.2	3.4 ±2.1	P = 0·02*
FT3 (pg/mL)	6.9 ± 1.5	3.1 ± 0.8	P < 0·01*
FT4 (pmol/L)	54 ± 11	21 ± 8	P < 0·001*
Creatinine (mg/dl)	0.3 ±0.1	1.1 ± 0.2	P < 0·03*
eGFR (MDRD)	127 ±37	101 ± 11	P < 0·01*

Correlation coefficients (r) between TSH, FT3, and FT4 levels, and renal function parameters were also calculated to determine if there is any association. TSH had a significant *weak* positive correlation with serum creatinine (r=0.452, p=0.016) and a significant moderate negative correlation with eGFR (r=-0.687, p=0.03) in all patients with thyroid dysfunction (Table 3).

**Table 3:** Correlation between thyroid function tests and creatinine in all participants TSH: thyroid-stimulating hormone; FT: free thyroxine; eGFR: glomerular filtration rate

	TSH		FT3		FT4	
	r value	p-value	r value	p-value	r value	p-value
Creatinine	0.452	0.016*	−0.088	0.683	−0.087	0.617
eGFR	- 0.687	0.03*	0.325	0.341	0.835	0.256

## Discussion

It is well recognized that thyroid and renal function interact, and that thyroid disease can significantly alter kidney function, particularly by affecting GFR [6,7]. In routine clinical practice, a variety of kidney function tests are used; the most often employed biomarkers are serum creatinine and eGFR. The current study's objective was to determine whether thyroid condition had any impact on the laboratory markers of renal function. Biochemical indicators of renal function in patients with thyroid dysfunction were assessed and compared before and after thyroid disorder treatment. According to the current study, there are notable variations in renal function that are correlated with the degree of thyroid dysfunction. Whether the renal function is represented as creatinine levels or as eGFR, these changes remain the same. Our study demonstrated a statistically significant reduction in the average serum creatinine level in the hypothyroid patients after treatment compared to before treatment whereas the mean eGFR significantly improved after treatment. Moreover, the average serum creatinine level in the hyperthyroid patients was significantly lower before treatment compared to after treatment, whereas the mean eGFR significantly dropped after treatment. TSH had a significant positive correlation with serum creatinine and a significant negative correlation with eGFR in all patients with thyroid dysfunction. Serum creatinine increases while eGFR decreases in hypothyroidism whereas serum creatinine decreases and eGFR increases in hyperthyroidism. These results are in accordance with previous research [2,7,8]. An increase in serum creatinine was linked with an increase in TSH levels. A reversible rise of serum creatinine in people with hypothyroidism has been linked in some earlier studies [3,8]. Even while earlier studies were conducted using less sensitive TSH and FT4 tests, they nonetheless produced results that are comparable to those recently described. Verhelst et al. showed that patients with hyperthyroidism had lower serum creatinine levels, while patients with hypothyroidism had higher values [9].

The GFR is reversibly decreased (by roughly 40%) in more than 55% of hypothyroid people. There are several mechanisms for the GFR decline seen in hypothyroid people. Reduced cardiac output, increased peripheral vascular resistance, intrarenal vasoconstriction, decreased renal response to vasodilators, and decreased expression of renal vasodilators like vascular endothelial growth factor insulin-like growth factor-1, all contribute to the reduction of renal blood flow in hypothyroidism. Reduced renal blood flow may also be caused by pathologic modifications to the glomerular structure in hypothyroidism, such as thickening of the glomerular basement membrane and expansion of the mesangial matrix. GFR is lost as a result of decreased sensitivity to adrenergic stimulation, decreased renin release, decreased angiotensin II, and diminished renin-angiotensin system action [2]. Due to renal parenchymal growth retardation in hypothyroidism, there is a structural constraint imposed by limited glomerular surface area for filtration. Additionally, there is decreased proximal tubular absorption of sodium, chloride, and water. The renal basolateral chloride channel expression is also decreased. As a result, decreased chloride reabsorption causes an increase in distal chloride supply, which in turn activates tubuloglomerular feedback mediated by the macula densa and lowers renin-angiotensin system activity. As a result, the GFR decreases [1,,2].

Myopathy and rhabdomyolysis are common mechanisms involved in the reversible elevation of serum creatinine detected in thyroid-associated kidney derangements. Normalizing the thyroid function through effective treatment usually results in normalizing the renal function [2,3]. In the current study, changes in renal function in hypothyroid patients tended to normalize after thyroid dysfunction is corrected. These results are in accordance with previous research, which stated that GFR levels in myxedematous patients are about a third lower than euthyroid values and are easily reversed on thyroxin replacement therapy [1,2].

Hyperthyroidism, on the other hand, has the opposite effect on serum creatinine and eGFR [2]. The causes are numerous and complex. First, due to its beneficial chronotropic and inotropic effects on the heart, hyperthyroidism raises cardiac output levels. Additionally, the activation of the renin-angiotensin system results in an increase in blood volume, which also increases renal blood flow. Overproduction of thyroid hormone lowers afferent glomerular arterioles' resistance, which raises glomerular hydrostatic pressure and GFR. By triggering tubuloglomerular feedback, a decrease in chloride load is brought on by an increase in chloride absorption in the proximal tubule and Henle's loop segments, which leads to an increase in GFR [2]. The basolateral sodium-calcium exchanger is fed by the rise in basolateral sodium content. These effects are reversed by treating hyperthyroidism, and the GFR returns to normal. In addition to an increase in GFR and a decrease in overall muscle mass, hyperthyroid patients' serum creatinine levels, an inverse marker of GFR, are much lower [10].

Given all of these observations, the effect of thyroid hormone on GFR can be used to explain variations in serum creatinine seen in different thyroid diseases. Although modest, these variations can matter to particular patients. Due to GFR alterations in hypothyroidism, patients receiving concurrent treatment with medications with limited therapeutic ranges (such as digoxin or metformin) may experience toxicity. Another important clinical implication of our study is that in patients with unexplained increases in serum creatinine, thyroid hormone screening is necessary to identify hypothyroidism.

Strengths and limitations

The main strength of this study is that it assesses the effect of thyroid dysfunction in both hypo- and hyperthyroidism before and after treatment. The main limitation of this study is the relatively small sample size and the single ethnic population. Secondly, we included some patients with mild diseases. Thirdly, for practical reasons, the thyroid antibody status was not analyzed and thus could not be investigated as a mediating factor. Also, due to the small number of patients with subclinical hypothyroidism, we could not perform subgrouping for this category. Lastly, due to the different methods used for treatment, the time frame for normalization was different among our patients. 

## Conclusions

Thyroid dysfunction is associated with deranged kidney function. There is a statistically significant reduction in the average serum creatinine level in the hypothyroid patients after treatment compared to before treatment whereas the mean eGFR significantly improved after treatment. Additionally, the average serum creatinine level in the hyperthyroid patients was significantly lower before treatment compared to after treatment, whereas the mean eGFR significantly dropped after treatment. TSH had a significant positive correlation with serum creatinine and a significant negative correlation with eGFR in all patients with thyroid dysfunction. It is crucial for the clinician to be aware of the link between thyroid disorders and aberrant biochemical markers of renal function to consider a thyroid function test when treating a patient whose biochemical markers of renal function are only mildly elevated. These findings also emphasize the need for monitoring creatinine in patients with thyroid dysfunction. The potential negative effects of thyroid dysfunction on renal function, however, require further research.
